# Immunofluorescence Assay for Serologic Diagnosis of SARS

**DOI:** 10.3201/eid1003.030493

**Published:** 2004-03

**Authors:** Paul K. S. Chan, King-Cheung Ng, Rickjason C. W. Chan, Rebecca K. Y. Lam, Viola C. Y. Chow, Mamie Hui, Alan Wu, Nelson Lee, Florence H.Y. Yap, Frankie W. T. Cheng, Joseph J. Y. Sung, John S. Tam

**Affiliations:** * Faculty of Medicine, Chinese University of Hong Kong, Shatin, New Territories, Hong Kong Special Administrative Region, People’s Republic of China

**Keywords:** coronavirus, diagnosis, serology, sensitivity, specificity, severe acute respiratory syndrome, SARS

## Abstract

We evaluated a virus-infected cell-based indirect immunofluorescence assay for detecting anti–severe acute respiratory syndrome-associated coronavirus (SARS-CoV) immunoglobulin (Ig) G antibody. All confirmed SARS cases demonstrated seroconversion or fourfold rise in IgG antibody titer; no control was positive. Sensitivity and specificity of this assay were both 100%. Immunofluorescence assay can ascertain the status of SARS-CoV infection.

On March 12, 2003, the World Health Organization (WHO) issued a global alert on outbreaks of atypical pneumonia (*1*). Cases were observed in Vietnam, Hong Kong, Singapore, and Toronto. As of June 2003, a total of 29 countries had been affected (*2*). WHO refers to this highly infectious disease as severe acute respiratory syndrome (SARS) and has formulated case definitions for surveillance (*3*). The virus causing SARS was identified in late March (*4*–*6*). The full genome of a few strains of the SARS-associated coronavirus (SARS-CoV) was soon available; it was confirmed to be a novel virus phylogenetically distinct from previous known coronaviruses (*7*,*8*). Since the discovery of SARS-CoV, laboratory diagnosis for the infection has become an important part of patient management, contact tracing, and epidemiologic study. In general, serology is the mainstay for ascertaining viral infection status. We report the evaluation of a first-generation assay based on the indirect immunofluorescence technique for detecting anti-SARS-CoV immunoglobulin (Ig) G antibody.

## The Study

We conducted this study at the teaching hospital of the Chinese University of Hong Kong, Prince of Wales Hospital, where a major outbreak of SARS had occurred (*9*). Patients admitted with clinical features suggestive of SARS were investigated for SARS-CoV infection by a combination of methods including direct detection of viral RNA by reverse-transcription polymerase chain reaction (RT-PCR) using primers COR1/COR2 (*10*), virus isolation using African green monkey kidney (Vero) cells, and serology. All RT-PCR–positive results were confirmed by repeat testing from the original sample; isolation positive results were confirmed by detection of SARS-CoV RNA from culture supernatant by RT-PCR.

### True SARS Cases

For the purpose of study analysis, a patient was defined as a true SARS case when he or she had the following two conditions: 1) fulfilled the WHO criteria of a probable case of SARS (*11*), and 2) had one or more specimens positive for SARS-CoV by RT-PCR, isolation, or both. From March to May 2003, we identified 128 patients who fulfilled our definition of a true SARS case. Sixteen of them died before a convalescent-phase blood sample could be collected; 9 received convalescent-phase plasma therapy. These 25 cases were excluded. As a result, 103 true SARS cases were analyzed. Three were pediatric patients of ages 5, 11, and 16 years. Eighty-six were adults from 21 to 64 years of age (mean 35.7, SD 11.3), 60.5% were female. The remaining 14 were elderly patients 66 to 89 years (mean 75.6, SD 7.8), 50.0% female. Pneumonia developed in all these patients; five required intensive care and eventually recovered; four died of the infection.

### Non-SARS Controls

Patients admitted to the Prince of Wales Hospital during 2000 for respiratory tract infections or febrile illnesses were used as non-SARS controls. The convalescent-phase serum samples that had been collected from these patients for viral and atypical pneumonia serologic screening were retrieved for this study. This control group consisted of 540 patients; 126 were pediatric patients 6 months to 15 years of age (mean 7.4, SD 3.1); 40.0% were girls. Of the 308 adults ages 16–65 years (mean 45.6, SD 10.3); 35.3% were female. For the 106 controls 65–86 years of age (mean 73.2, SD 3.7), 65.0% were female. Overall, 16.3% of this control group were confirmed to have infections with respiratory viruses or atypical pathogens.

In addition to hospitalized patients, a healthy group was included as non-SARS controls. This group comprised 635 medical students 19–31 years of age (mean 23.5, SD 2.2); 41.9% were female. Their blood samples, which had been submitted for pre-varicella zoster virus vaccination screening in 2000, were retrieved for this study.

### Antibody Detection

Anti-SARS-CoV IgG antibody was detected by the indirect immunofluorescence technique. Vero cell monolayer at 90% confluence was inoculated with SARS-CoV. The coronavirus stock used was the third passage of an isolate grown from a SARS patient. The full genome sequence of this isolate has been published (GenBank accession no. AY278554). Infected cells were harvested when cytopathic effect was observed on 70% of the cell monolayer. With our laboratory conditions, this event occurred consistently at 96 to 100 hours after virus inoculation. Infected cells were mixed with noninfected Vero cells at a ratio of 1 to 1. After being washed three times with phosphate-buffered saline (PBS), cells were spotted onto 12-well, Teflon-coated glass microscope slides. The slides were allowed to air-dry and then fixed for 10 minutes with 100% pre-chilled acetone, and stored at –70°C until use.

Serum samples were heat-inactivated at 56°C for 30 minutes and then diluted in PBS. An aliquot of 25-μL diluted serum sample was placed on a coated well and incubated at 37°C for 30 minutes in a moist chamber. After being washed three times with PBS, a fluorescein isothiocyanate–conjugated rabbit anti-human IgG antibody (Dako, Denmark) was added at a dilution of 1 to 40, and incubated for 30 minutes at 37°C. In each test run, a positive control serum with known titer was tested in twofold serial dilutions to guard the sensitivity, and results were crosschecked by two experienced technicians.

Our diagnostic approach was to perform a screening test at a dilution of 1 to 40 for serum samples collected at >10 days after the onset of illness. Upon special circumstances, testing might be performed on earlier samples. When the screening result was positive, a follow-up test at twofold serial dilutions starting from 1 to 40 was performed together with the corresponding acute-phase serum sample. On the other hand, if the screening result was negative or showed nonspecific fluorescent signals, a follow-up sample was collected for repeat testing. In addition, when a titer of 1:40 was obtained on the second sample, a third sample was collected for repeat testing. A seroconversion or fourfold rise in antibody titer was regarded as serologic evidence of recent SARS-CoV infection.

The serologic data of the true SARS cases used for this analysis were based on the results obtained from our routine test runs. During the outbreak, our laboratory-performed screening test are conducted on every alternate day, and follow-up titration tests occur the next day. The samples included in each test run were based on clinicians’ requests containing a variable proportion of cases that turned out to be non-SARS; the technicians did not know the results of other SARS investigations for the testing samples. For the non-SARS controls, each test run contained 50 testing samples mixed with 5 known positive controls and was tested in a blind fashion.

## Conclusions

A total of 212 serum samples from the 103 true SARS cases were tested for anti–SARS IgG antibody. Four samples (1.9%) showed fluorescent signals from all cells fixed on the slide. Since we had mixed infected cells with an equal amount of noninfected cells, we expected to observe genuine positive signals from approximately 50% of cells fixed on the slide. Therefore, these four samples were regarded as nonspecific. Follow-up samples were obtained from these patients, and all tested positive. Overall, 94 (91.3%) cases showed seroconversion, and 9 (8.7%) showed a fourfold rise in antibody titer. The positive rate and antibody titer with respect to the time of specimen collection are shown in the Table. We detected the earliest seroconversion on day 6 after the onset of fever. The antibody-positive rates for samples collected during days 5–10, 11–15, and 16–20 after the onset of fever were 34.3%, 78.3%, and 97.7%, respectively.

**Table:** Anti-SARS-CoV IgG positive rate and titer according to time of blood sample collection^a^

Time of sample collection after onset of fever	No. of samples tested	No. (%) of samples with anti–SARS IgG antibody detected	Anti-SARS IgG antibody titer, range (mode)
1–5 days	64	0	–
6–10 days	35	12 (34.3)	40–320 (160)
11–15 days	23	18 (78.3)	40–640 (320)
16–20 days	43	42 (97.7)	40–2,560 (640)
21–37^b^ days	47	47 (100)	80–5,120 (640)

^a^SARS, severe acute respiratory syndrome; CoV, coronavirus; IgG, immunoglobulin G; –, not applicable. ^b^Median collection time: 22 days; interquartile range: 4 days

Of the 1,175 samples obtained from the non-SARS control groups, 24 (2.0%) showed fluorescent signals from all cells fixed on the slide. These samples were regarded as nonspecific. The remaining 1,151 samples were negative for anti–SARS IgG antibody.

Serologic diagnosis remains an indispensable means for confirming viral infection status. Antibody assays based on virus-infected cells or whole viral lysate might produce cross-reactivity between infections because of closely related viruses. As common cold–associated coronavirus infections are highly prevalent, the specificity of whole virus-based assays for the diagnosis of SARS-CoV infection is a concern. Our results indicated that an infected cell-based indirect immunofluorescence test for anti-SARS IgG antibody provided a sensitivity and specificity of 100%. However, this immunofluorescence test is relatively labor intensive. Experienced technicians are required to examine the results, in particular to differentiate nonspecific signals from positive results. These properties make the test not ideal for large-scale studies. Nevertheless, its high sensitivity and specificity make the test applicable to ascertain infection status and to serve as a reference for assessing the performance of high-throughput second-generation assays such as enzyme immunoassay. The immunofluorescence test can also be used as a confirmatory assay for samples reactive to screening assays. Further development of more feasible assays with high throughput and performance should be pursued. Evaluation of the role of other classes of anti–SARS-CoV antibodies in the diagnosis of SARS-CoV infection is needed.
